# Supplementary material for: Dynamical SPQEIR model assesses the effectiveness of non-pharmaceutical interventions against COVID-19 epidemic outbreaks
Source: PLoS One. 2021 May 21;16(5):e0252019. doi: 10.1371/journal.pone.0252019 (PMC8139462; doi:10.1371/journal.pone.0252019)
Supplement: S1 Text — (PDF) [file pone.0252019.s002.pdf]

S1 File:
"Dynamical SPQEIR model assesses the effectiveness of non-pharmaceutical interventions against COVID-19 epidemic outbreaks"

Daniele Proverbio[1], Françoise Kemp[1], Stefano Magni[1], Andreas Husch[1], Atte Aalto[1], Laurent Mombaerts[1], Alexander Skupin[1], Jorge Gonçalves[1], Jose Ameijeiras-Alonso[2], and Christophe Ley[3]

[1]University of Luxembourg, Luxembourg Centre for Systems Biomedicine
[2]KU Leuven, Department of Mathematics
[3]Ghent University, Department of Applied Mathematics, Computer Science and Statistics

Contents

1 Effects of τ on epidemic curves properties 1
1.1 Combining τ and ρ . . . 1
1.2 Combining τ and μ . . . 2
1.3 Combining τ and χ' . . . 2
1.4 Combining τ and η . . . 3

2 Discussion on model fitting 4
2.1 Discussion on least-squares fitting . . . 4
2.2 Parameter space characterization by means of Markov Chain Monte Carlo . . . 4

1 Effects of τ on epidemic curves properties

In the main text, we focused the conceptual analysis on the sensitivity of epidemic dynamics on mitigation parameters. However, it is well known that early adoption of interventions is also important [1]. In the SPQEIR model, the delay in applying interventions is modeled by an extra parameter τ. In this section, we analyse its impact on qualitative and quantitative properties of epidemic curves.
We observe that, for all mitigation parameters, τ does not significantly alter the qualitative properties of epidemic dynamics: their corresponding diagrams "height of peak vs param" is maintained, as well as the trend of mitigation timing. Instead, τ affects their quantitative aspects: the height of the peak before mitigation and the values of mitigation timing $\mathbb{T}$. Overall, this justifies the use of an arbitrary value for τ for the conceptual analysis (Sec. 3.1 in Main Text), whereas it was properly estimated for each country when the fit was performed (Sec. 3.3 in Main Text).

1.1 Combining τ and ρ

Different delays in issuing interventions governed by the parameter ρ display the same diagram "height of peak vs ρ", with the same critical value of the parameter $\rho = 0.4$ that yields mitigation (Fig. 1). Instead, τ modifies the peak value that is reached before mitigation, for $\rho < 0.4$. When considering the mitigation timing, Fig. 1 (Right) shows that its non-monotonous trend is overall not altered by τ. However, the maximum time decreases, since longer delays are associated to a higher depletion of the

Susceptible pool. On the other hand, the optimal value for minimising the mitigation time becomes smaller, meaning that stronger measures are required to flatten the curve in minimal time.

This analysis implies that, while qualitative characteristics associated to the epidemic dynamics are conserved, quantitative values change under the influence of τ, that should therefore be taken into account when studying real-world scenarios.

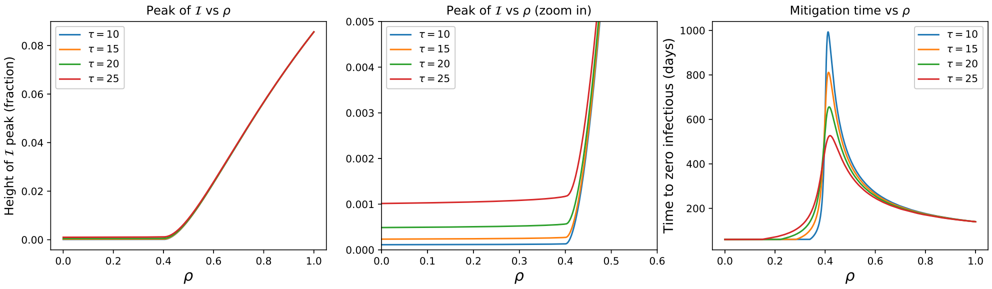

Fig. 1 (Left) Global dependence of the peak of daily cases on ρ, for different τ values. (Center) A closer look on the infectious peak, depending on ρ values associated to mitigation and on delays τ. Although the qualitative trend is maintained, the peak values change for different τ. (Right) Mitigation timing depending on ρ, for different τ. To minimise the timing, a smaller τ can be coupled with higher ρ, whereas longer delays require smaller ρ values to be as efficient.

1.2 Combining τ and μ

When mitigation is pursued after acting on those measures associated to the parameter μ, time delays are associated with higher peaks of the infectious curve (Fig. 2 Left and Center), but the trend is conserved. The mitigation timing might be slightly reduced when small μ values are active but, for stronger protection rates, it is significantly decreased by prompt interventions (Fig. 2 Right).

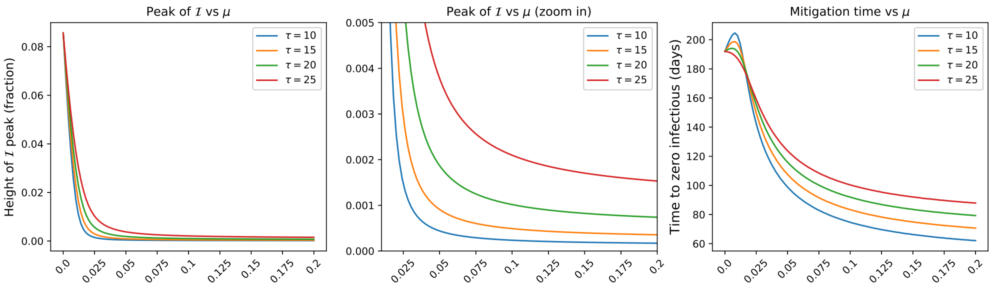

Fig. 2 (Left) Global dependence of the peak of daily cases on μ, for different τ values. (Center) A closer look on the infectious peak, depending on μ values associated to mitigation and on delays τ. The qualitative, monotonous trend is maintained, but the peak values change for different τ. (Right) Mitigation timing depending on μ, for different τ. For small μ values, a longer delay could yield faster mitigation because of the faster depletion of the Susceptible pool, but the trend is soon inverted for higher μ values.

1.3 Combining τ and χ'

This section considers the removal of E individuals from the epidemic system, for instance because of quarantining after contact tracing. In the SPQEIR model, this strategy is modelled by the parameter χ' (see Main Text for discussion). Longer delays in issuing such interventions do not alter the global features of the bifurcation diagram, but are associated with higher peak values (Fig. 2 Left and Center). Longer delays are also associated with faster mitigation timing, due to the fact that more people have the chance to develop the infection. The trade-off is resolved when employing the most effective measures (maximum χ'), which yields minimal peak height as well as shortest mitigation timing.

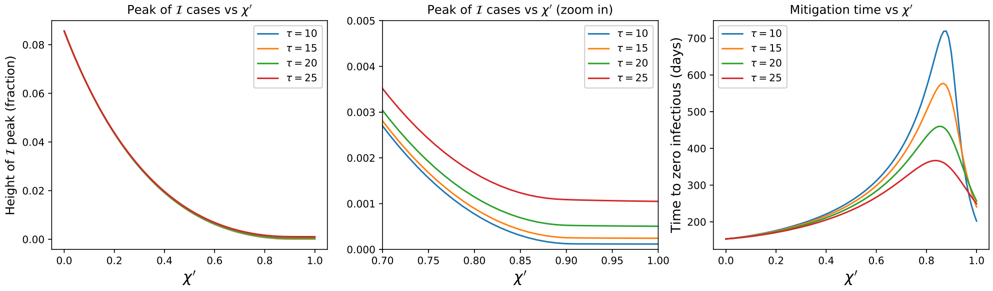

Fig. 3 (Left) Global dependence of the peak of daily cases on χ', for different χ' values. (Center) A closer look on the infectious peak, depending on χ' values associated to mitigation and on delays τ. The bifurcation diagram is maintained, but the peak values after mitigation change for different τ. (Right) Mitigation timing depending on χ', for different τ.

1.4 Combining τ and η

In the SPQEIR model, further removal of Infectious individuals (e.g. for tracking and isolation) is parameterised by η. As for other parameters, the qualitative behavior of the epidemic dynamics, when driven by η, remains unaltered by extra delays τ. As shown in Fig. 4, the bifurcation diagram remains consistent, even though the quantitative values of mitigated peaks are higher for higher τ. However, contrariwise with what observed for other parameters, delays in issuing measures slightly change the critical η values that are necessary to achieve mitigation (Fig. 4, Center), that are lowered for higher τ. This is a byproduct of having let the virus spreading more in the population and resulting in a higher initial peak. In addition, longer delays correspond to overall longer mitigation timing for same η (Fig. 4, Right). This expands what discussed e.g. in [2], further stressing the importance of prompt isolation of infectious individual for epidemic management and control.

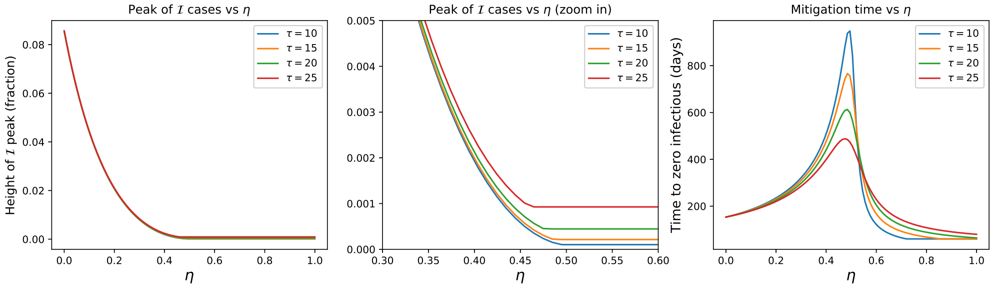

Fig. 4 (Left) Global dependence of the peak of daily cases on η, for different τ values. (Center) A closer look on the infectious peak, depending on η values associated to mitigation and on delays τ. The bifurcation diagram is maintained but the critical value is slightly shifted. Peak values after mitigation change for different τ. (Right) Mitigation timing depending on η, for different τ.

2 Discussion on model fitting

As discussed in the Main Text, we fitted the SPQEIR model on several countries to show that such minimal model can reproduce epidemic curves and enable further explanation of the impact of several non-pharmaceutical interventions. However, the structure of the model retains a certain level of degeneracy among its parameters, which lead to identifiability limitations that are known in SIR-like models [3]. In particular, several parameter combinations could yield to similar values of the control parameter $\hat{R}$. In this section, we further discuss the relevance of the *lmfit* fitting presented in the Main Text and we compare and support it by discussing the results of a Bayesian inference approach based on a Markov Chain Monte Carlo (MCMC) scheme for model fitting, that allows us to fully explore the parameter space and relevant combinations of the parameters. This procedure enables a detailed analysis of the identifiability of our parameters and justifies the subsequent use of a least-square methodology to estimate a single, reasonable set of parameters that could well explain the empirical data.

2.1 Discussion on least-squares fitting

As explain in the Main Text, we first fitted the SPQEIR model with a single free parameter, to get a meaningful value for $\hat{R}$ (the "only social distancing" setting). Then, we fitted the full model, with country-specific parameters (*cf.* Table 1), making sure that analogous $\hat{R}$ values were obtained. We recall that not all parameters were allowed to be free, but only some were applied, i.e. those that can be matched with a corresponding measure documented to have been applied in the real country. Although possibly not unique, the identified parameter values are the most likely after gradient descent [4] and yield to non-negligible numbers of individuals being affected by the corresponding measures. Table 1 reports the cumulative numbers of individuals that flowed to Q and P compartments during the first intervention period, marked in grey in Fig. 9 of the main text, due to fitted parameter values (also reported in Fig. 9 of main text). These estimates are useful, as order of magnitudes, to appreciate the effect of single, targeted interventions coming from different non-pharmaceutical interventions, in addition to population-wide social distancing. Further verification and precise assessment of such values are demanded to future, complementary studies that make use of welfare partner data.

Country	Quarantined	Protected
Austria (AT)	430	63000
Denmark (DK)	2500	120000
Ireland (IR)	4000	730000
Israel (IL)	2200	63000
Lombardy (LO)	3100	69000
Switzerland (CH)	520	59000

Table 1: Total numbers of individuals flowing to outer compartments of the SPQEIR model, during the considered time period (grey area in Fig. 9 of main text), under the action of fitted parameters. Following the discussion about parameter identifiability, these numbers are meaningful as orders of magnitude, and are thus reported with rounding on the second significant digit.

2.2 Parameter space characterization by means of Markov Chain Monte Carlo

We next verified the constraints on model paramters and the degeneracy between them with an additional analysis, based on Bayesian inference implemented by means of a Markov Chain Monte Carlo (MCMC) approach, which allows to further identify the individual parameters values and the constraints on their combinations in the parameter space, as well as the associated uncertainties. For the current MCMC analysis, we employed as likelihood function a commonly used sum of square residuals (SSR), which measures the distance between the model simulation and the data as:

$$SSR\left(\overline{\tau}|data\right) = \sum_{i=T_0}^{T_f} \left[C^{data}\left(t_i\right) - C^{model}\left(t_i, \overline{\tau}\right)\right]^2 . \quad (1)$$

In Eq. 1, $\overline{\tau}$ is the array of parameters representing measures relevant for that country, $t_i \in \{T_0, T_0 + 1, \ldots, T_f - 1, T_f\}$ indicates the number of days from the beginning of the epidemic (assumed for that country), T_0 and T_f are respectively the first and last day under investigation, C^{data} indicates data of active infections and C^{model} to the corresponding model quantity. The SSR is related to the reduced χ^2, both being ways to measure the geometric distance between model simulation and data.

The applied sampling technique is the Delayed Rejection Adaptive Metropolis (DRAM) scheme [5], from the python package *pymcmcstat* [6]. We run 8 chains in parallel, initialized at random initial conditions. Each chain has 50000 iterations, which ensure the convergence of the chain. For the Bayesian steps, the simplest non-informative flat priors probability distributions are assumed on each parameter. These are chosen as there is no need to incorporate additional information on the parameter values, except for the boundaries of the intervals which are allowed. Furthermore, flat priors do not introduce any further bias towards a particular parameter set. The parameter intervals to be explored are chosen to be reasonable with respect to the conceptual analysis: ρ ranges from 0.1 to 0.4, μ from 0.001 to 0.12, χ from 0.001 to 0.7 and η from 0.001 to 0.1. The chain convergence was assessed by the Gelman-Rubin diagnostic, which analyses the variance within and between each chain set. Results of the Bayesian fitting are reported below, and we repeated the procedure on several of the countries considered.

As mentioned in Main Text, we observe that several combinations of parameters lead to similar good agreement (measured by the SSR) between the model simulation and the data, and thus result in a high posterior probability (dark red color in the figures). Other combinations lead to poorer agreement between model and data, and are thus associated to low posterior probability distribution (light red, blue or dark blue regions). In order not to overload the text, we report 3 example countries with different parameter sets. Others are analogous and can be reconstructed with the shared code. The figures below (Figs. 5 for Austria, 6 for Ireland and 7 for Switzerland) showcase which areas of the parameter space have a higher posterior probability, and thus indicate which combinations of parameters would be more likely to provide a good fit between model and data. The figures are reconstructed by marginalising the full distribution from MCMC chains over couples of parameters at a time. In fact, some parameter pairs are correlated, and a certain degree of degeneracy exists between parameters, in the sense that often simultaneous changes in two parameters would equally well allow the model to fit the data. This highlights the identifiability issues discussed in the Main Text, namely that multiple sets of parameter values could be employed resulting in similar fit of the model to the data. Eventually, to provide one plausible choice, we reported in the Main Text the best gradient descent fit obtained with *lmfit* scheme.

References

[1] Deutsche Gesellschaft für Epidemiologie. Stellungnahme der Deutschen Gesellschaft für Epidemiologie (DGEpi) zur Verbreitung des neuen Coronavirus (SARS-CoV-2). *www.dgepi.de*, 2020.

[2] Xiuli Liu, Geoffrey JD Hewings, Minghui Qin, Xin Xiang, Shan Zheng, Xuefeng Li, and Shouyang Wang. Modelling the situation of COVID-19 and effects of different containment strategies in China with dynamic differential equations and parameters estimation. *Available at SSRN 3551359*, 2020. doi: http://dx.doi.org/10.2139/ssrn.3551359.

[3] Weston C. Roda, Marie B. Varughese, Donglin Han, and Michael Y. Li. Why is it difficult to accurately predict the COVID-19 epidemic? *Infectious Disease Modelling*, 5:271 – 281, 2020. ISSN 2468-0427. doi: https://doi.org/10.1016/j.idm.2020.03.001. URL http://www.sciencedirect.com/science/article/pii/S2468042720300075.

[4] Matthew Newville, Till Stensitzki, Daniel B. Allen, and Antonino Ingargiola. LMFIT: Non-Linear Least-Square Minimization and Curve-Fitting for Python, September 2014. URL https://doi.org/10.5281/zenodo.11813.

[5] Heikki Haario, Marko Laine, Antonietta Mira, and Eero Saksman. Dram: efficient adaptive mcmc. *Statistics and computing*, 16(4):339–354, 2006.

[6] Paul R Miles. pymcmcstat: A python package for bayesian inference using delayed rejection adaptive metropolis. *Journal of Open Source Software*, 4(38):1417, 2019.

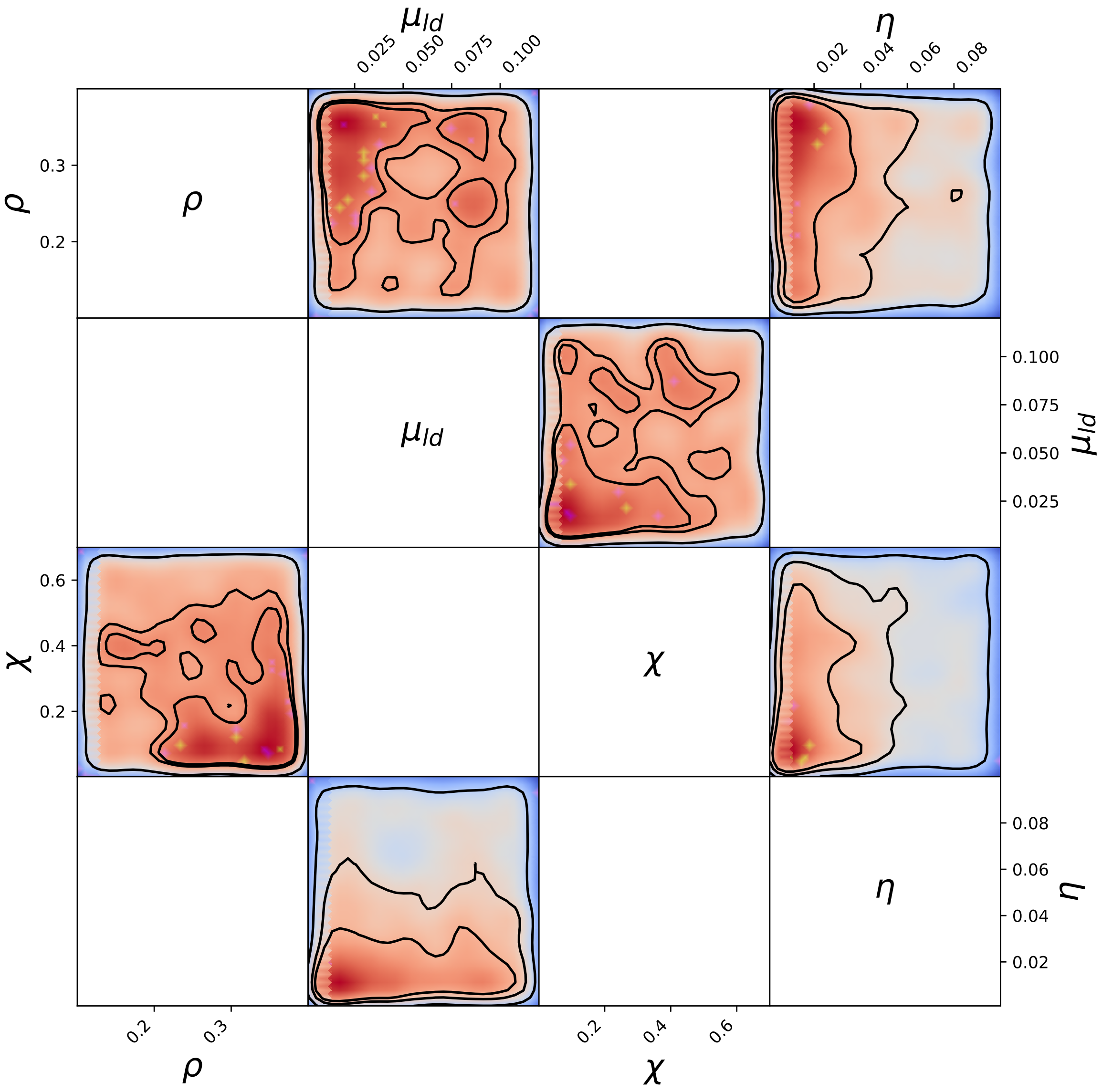

Fig. 5 Estimated posterior probability distribution from Markov Chain Monte Carlo projected over each couple of parameters, for Austria. Each square shows 2D projection of the posterior over two parameters. The parameter names are reported at the margins and on the diagonals. Each 2D projection is reported only once once as they are symmetric, thus the white squares. The posterior probability is represented by the nuances of red towards blue, with red being high probability and blue being low (probability close to 0). The three black contours correspond to 25%, 50% and 90% Bayesian credible regions.

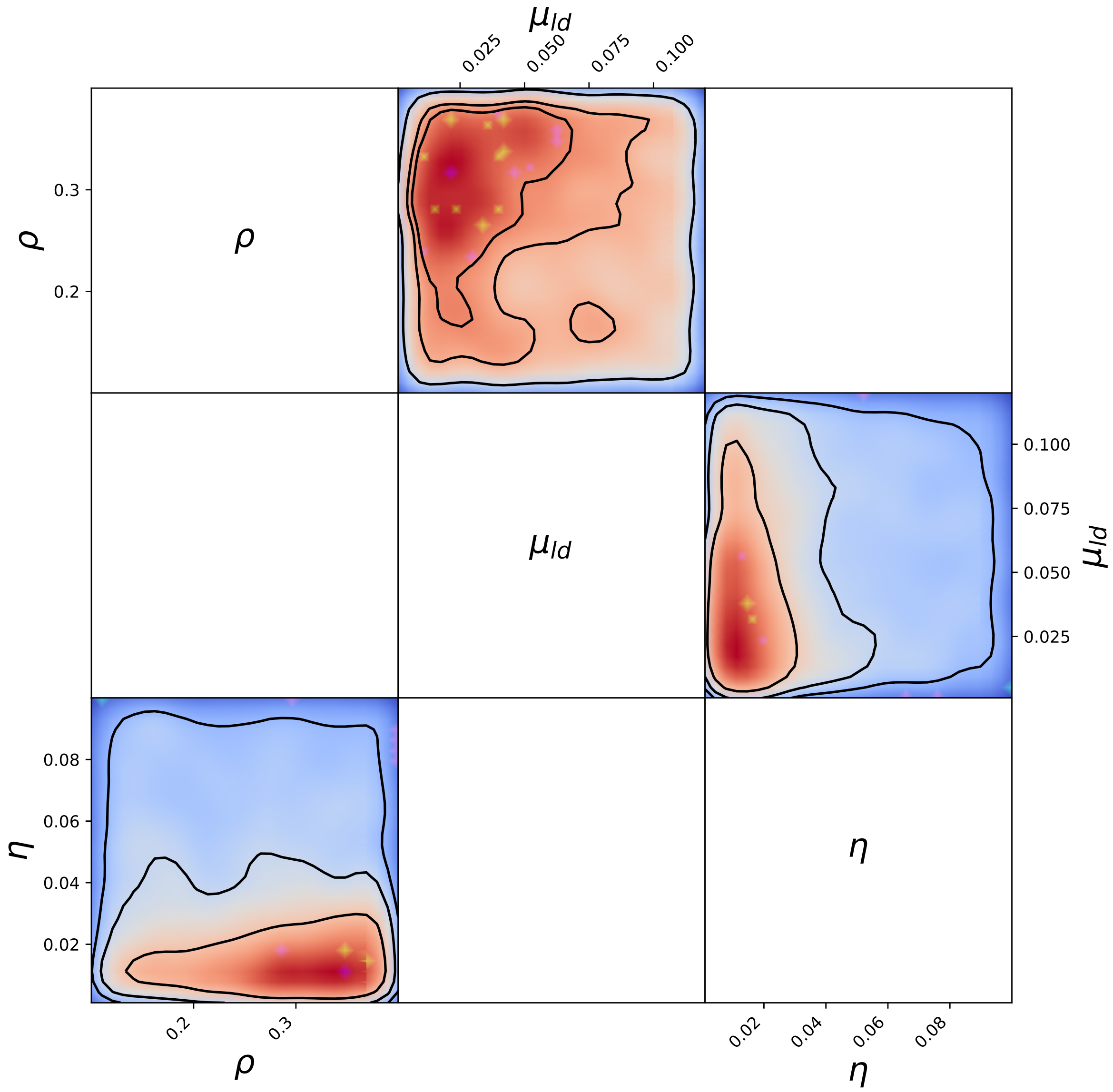

Fig. 6 Estimated posterior probability distribution from Markov Chain Monte Carlo projected over each couple of parameters, for Ireland. Each square shows 2D projection of the posterior over two parameters. The parameter names are reported at the margins and on the diagonals. Each 2D projection is reported only once once as they are symmetric, thus the white squares. The posterior probability is represented by the nuances of red towards blue, with red being high probability and blue being low (probability close to 0). The three black contours correspond to 25%, 50% and 90% Bayesian credible regions.

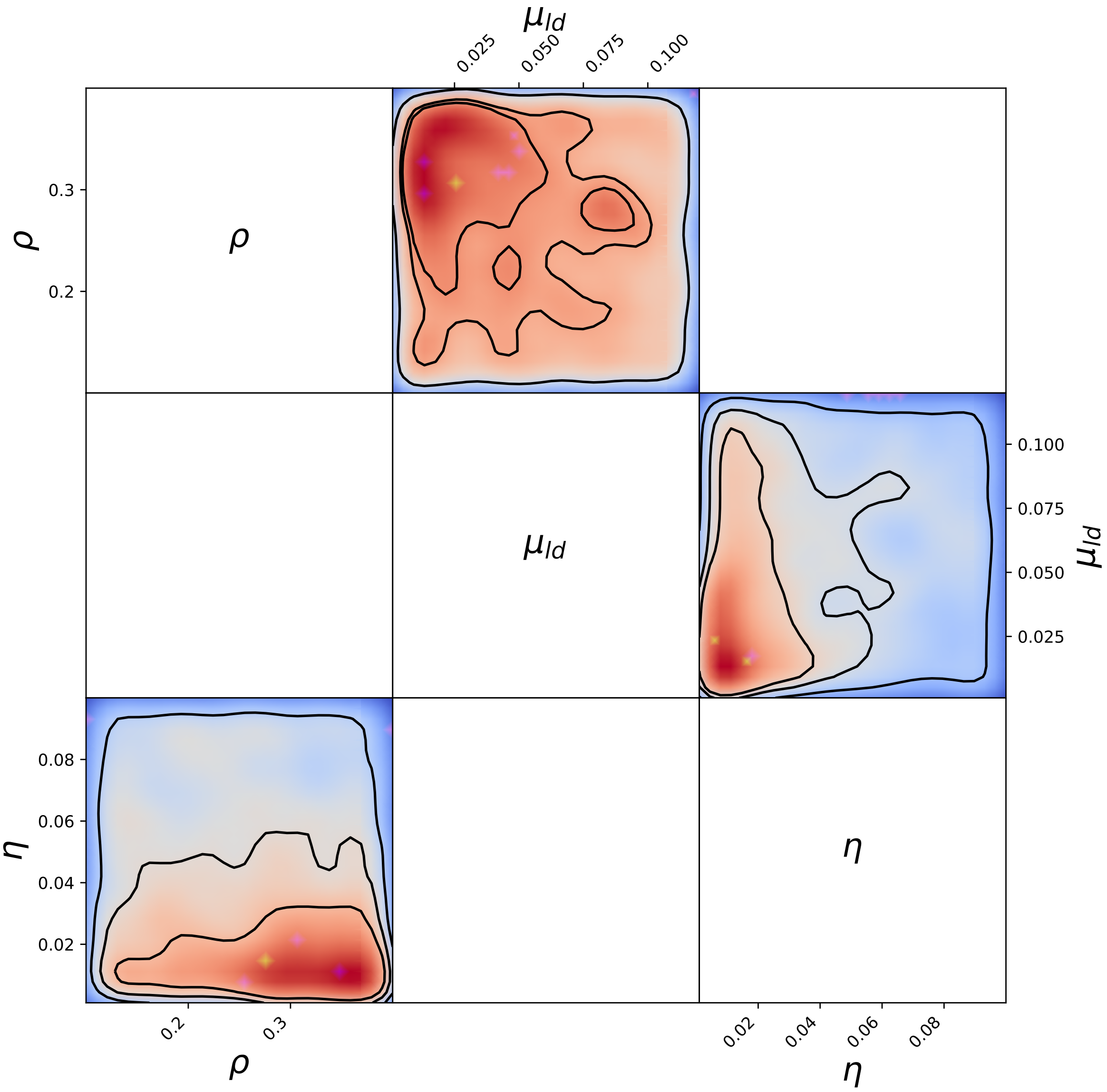

Fig. 7 Estimated posterior probability distribution from Markov Chain Monte Carlo projected over each couple of parameters, for Switzerland. Each square shows 2D projection of the posterior over two parameters. The parameter names are reported at the margins and on the diagonals. Each 2D projection is reported only once once as they are symmetric. The posterior probability is represented by the nuances of red towards blue, with red being high probability and blue being low (probability close to 0). The three black contours correspond to 25%, 50% and 90% Bayesian credible regions.
