# Supplementary material for: Dynamical SPQEIR model assesses the effectiveness of non-pharmaceutical interventions against COVID-19 epidemic outbreaks
Source: PLoS One. 2021 May 21;16(5):e0252019. doi: 10.1371/journal.pone.0252019 (PMC8139462; doi:10.1371/journal.pone.0252019)
Supplement: Attachment — Submitted filename: Response to reviewers.docx [file pone.0252019.s003.docx]

Dear editor, dear reviewers,

We thank you for your careful review and for the list of suggested improvements. Please find below detailed responses to the points you raised. Our responses are in blue. In the main text, the parts we edited, following your suggestions, are in red. Please notice that we also added Fig. 7 and updated Fig. 8 and 9 (of the new version) according to what suggested by you and discussed in the answers below.

**Reviewer #1:**

The paper introduces in the SEIR model 3 different types of non-pharmaceutical interventions i.e. social distancing, quarantine of exposed individuals and generalized lock-down. After the outbreak of COVID pandemic several papers have been published in this direction e.g.

Das et al. medRXIV <https://doi.org/10.1101/2020.06.04.20122580>;
Tomas de-Camino-Beck. medRXIV <https://doi.org/10.1101/2020.05.19.20106492>;
Perkins et al. Bulletin of Mathematical Biology 82, 118 (2020);
Lai et al. Nature volume 585, 410–413 (2020);
Alrrashed et al Informatics in Medicine Unlocked 20, 100420 (2020)

and also for the SIR model where quarantine is directly introduced on the infected nodes:

Anand et al. Transactions of the Indian National Academy of Engineering 5, 141–148 (2020)
Giordano et al. Nature Medicine 26, 855–860 (2020)
Mancastroppa et al. Phys. Rev. E 102, 020301(R) (2020)

Many other papers have been recently published or submitted.

We updated out bibliography with recent publications, including some of the ones mentioned above [6, 7, 8, 11, 12, 16, 18, 35, 45, 49, 52, 55, 56, 57]. We also integrated our references with a discussion of relevance of epidemic control not only on the healthcare [17,18,31], but also on the economic system [32,33,34]. Additionally, we discussed the reliability of the empirical data used for model fitting. In fact, we payed particular attention that, across the analysed countries, the share of positive tests was similar [27] and no significant deviation from expected dynamics was observed by studies applying Benford’slaw [28, 29].

Regarding the vast recent modelling literature, several studies have deeply analyzed the effects of specific interventions in selected countries, by statistical methods on simple and interpretable SIR-like models [1, 2, 6, 7, 8, 16, 17, 18, 35, 44], while in our study we consider the plausible impact of a combination of multiple strategies, and the difference from applying single interventions. A similar analysis was done with complex and data-demanding agent-based models [3, 31], which are often less generic, since focusing mostly on one country at a time. Instead, our model provides generic results that can be valid over several countries as shown in Fig. 9. Other works focus on single interventions with stochastic models [45, 55, 59]; we complement them by a different perspective. Many papers are focused on developing future projections [17, 41, 42, 60], but our aim is to discuss how to obtain similar mitigation with different strategies, to inform future decisions. As pointed out by Reviewer#1 below, additional papers account for extra features of importance for pandemic modelling. We discuss this in our response below. We are aware of abundant literature that consider conceptual models and the effect of different parameters (either published [11,12] or as preprints, e.g. Chladnà, medRxiv, 2020). We believe that our additional comparison with empirical data (Fig. 9) can contribute to the discussion about the relevance of such abstract strategies in real-world settings. Finally, our choice of modelling and selection of empirical data is based on socio-political analysis reports (sec 2.2 and Table 1), thus incorporating a multi-disciplinary approach in our study.

In these models often several aspects are taken into account which are overlooked in the present paper e.g. hospitalization, asymptomatic individuals, heterogeneous behavior of the individuals. The authors should explain what is the main advance with respect the vast recent literature.

Our paper aims at complementing the recent literature by synthetizing several control strategies into a readily interpretable model and by assessing their efficacy both alone and in synergy. This way, we can discuss the merits of each strategy and the possibility of mitigating the curve by a combination of them. The paper focuses on the average effects of such strategies on the infectious curve. We ultimately show that analogous containment levels of the infectious curve can be achieved by alternative synergies of non-pharmaceutical interventions. Hence, policymakers could aim to achieve the desired mitigation by measures targeting different population groups. This synergistic approach still remains of high importance in these second/third waves, where countries need to balance different non-pharmaceutical interventions to keep the infection at bay, while containing the impacts on other aspects of society. Following the remarks by reviewers, and recent literature, we have included isolation of infectious individuals into the model and simulations, on top of isolation of latent carriers.

We acknowledge that there are aspects of the pandemic found in other studies that were overlooked here, such as hospitalization, asymptomatic individuals, and heterogeneous behaviour of individuals. While these are all important aspects that can be crucial to answer certain questions, they are not needed here to answer the main question of the present paper: how strategies mitigate the infectious curve. Asymptomatic/undetected cases do play a role on the infection curve, but estimating their numbers is affected by large uncertainties. Thus, we opted for a more conceptual model without explicit asymptomatic/undetected compartments. When we compare our framework to empirical data, our goal is mostly to show that the model can reproduce the realistic dynamics observed in real data across a multitude of countries, and that synergies of several measures applied with moderate intensity can still provide the same benefit than a much more intense application of one intervention only.

On the other hand, to address different questions that require hospitalizations and asymptomatic/undetected cases, our group carried out a distinct study that includes such additional aspects, plus deaths and vaccination, throughout several epidemic waves (<https://www.medrxiv.org/content/10.1101/2020.12.31.20249088v1.full>).

In this respect, the authors consider the simultaneous adoption of three different kinds of measures, however it is not clear in the paper what is the main advantages of introducing this modelization. In particular which are the different effects due to the three strategy of containment? E.g. when you consider the fit of datasets the use of a single strategy (i.e. social distancing) provides a very similar fit of the case where the synergy of the different strategies is considered; also the improvement in the X^2 is very small. So it is not clear what is the advantage of adopting the different strategies, in the description of non-pharmaceutical interventions.

The model links several control strategies to real-world intervention programs, to investigate the possibility of achieving similar mitigation effects by using different strategies. The possibility of choosing among several strategies is of practical importance for decision makers. The impact of said strategies on the infectious curve is investigated in the dedicated sections (3.1.1, 3.1.2, 3.1.3, 3.1.4) and in panels (a) of Fig. 2, 3, 5 and 7). Increasing social distancing delays and decrease the height of the peak of infections. Increasing active protection as well decreases the height of the peak of infections, but it anticipates the occurrence of such peak. Increasing active quarantining also delays and decrease the height of the peak of infections like social distancing, but the same decrease in height of the peak by active quarantining is associated with shorter delay than with social distancing. The effects of measures targeting S and E population groups is also compared with those of isolation of contagious individuals (3.1.4). This shows how much preventive measures can buffer imperfect isolation of I persons. While not described by the model, these strategies are also expected to potentially have different effects on the population at the economic, social and psychological levels.

In addition, the performed fitting illustrates how well we can explain the evolution of empirical infectious curves with different control strategies. The chi^2 illustrates that the epidemic curves from 6 countries could indeed be fitted equally well (similar X^2, only small changes) by either a strong “social distancing” intervention or by milder \rho and synergistic measures. This is clearer in Fig.9 of the new version, where we also consider the isolation of infectious individuals. Moreover, we observe a high degree of correlation among some parameters. This is informative about the advantage of adopting a synergy of interventions instead of just one (e.g. only social distancing), in that it achieves a similar mitigation of the infection curve with interventions that can be selected based on the goal of limiting the impact on economy and society. This analysis can thus inform policymakers about the effects of strengthening non-pharmaceutical interventions others than social distancing, as e.g. contact tracing, mass testing, rapid testing.

In summary the paper introduces a model with a description of three types of non-pharmaceutical interventions, this interventions have been already introduced in different papers even if with some differences. In this context, from a theoretical point of view the authors do not evidence any peculiar or interesting effect due to the presence of these three terms; while a comparison with datasets does not show any advantages in interpretations of the epidemic evolution in the different countries. In this perspective I think that the paper is not suitable for publication without a significant improvement.

We complement the analysis of other valuable works, by considering combinations of control interventions in a simple, easily interpretable SIR-like model. Some seminal works did so with Agent-based models (e.g. [31]) but they require large and variegate data sets and are often less generic, since focusing mostly on one country at a time. Here, we highlight a general finding, which we found occurring in multiple countries. Introducing new terms allows for clearer interpretation of the effect of distinct control strategies that can achieve the same mitigation as untargeted, population-wide social distancing. From our experience in the Luxembourg COVID-19 Taskforce, this is useful to balance different options in decision making. The key message of our study is that fostering, strengthening and empowering additional interventions (other than social distancing) can achieve the same mitigation on the infection curve. This is investigated both from a conceptual point of view (Fig. 8) and by considering the evolution of empirical data under mitigation strategies (Fig.9). In the latter, differences in rho might appear small, but in fact correspond to significant changes in people’s lifestyle in terms of measures employed to obtain them (curfew, home office, closure of restaurants, shops, recreational activities, sport clubs and ultimately lockdown). Changes in the second digit of \rho do correspond to significant changes in lifestyle for a considerable amount of people, see e.g. https://www.medrxiv.org/content/10.1101/2020.12.31.20249088v1.full.

Another very important remark is about the model for a generalized lock-down. In your approach only susceptible nodes are isolated in the state P while I expect that a fraction of the whole population in a lock-down is isolated; in particular I expect that the sudden isolation of infected and exposed individuals should have a larger effect than the isolation of susceptible ones. Moreover I think that the process should be described only by the approach in figure 4 i.e. a sudden isolation of a large number of individuals which remain isolated during the whole period of non-pharmaceutical interventions; while other individuals do not self isolates (e.g. they do an essential job), so that after the quick self isolation mu is restored to 0. On the other hand, a self isolation with constant rate, in the whole period of intervention seems to be very unlikely. An even a similar and simpler model of a generalized lock down could be at the beginning of the intervention an instantaneous isolation in the state P of a fraction of individuals (independently of their states). Therefore, I think that the different scenarios and the data fitting should take into account only the approach in figure 4 (clearly with possible different fraction of people that self isolate).

The approach described by Reviewer#1 was used for the fitting, in which a fraction of individuals (independently of their state) is isolated or disconnected through the action of all parameters together. Indeed, we used the mu_ld from Fig.4 for the fitting. Following Reviewer#1 comments, we edited the text to make the point clearer (“For the protection parameter, we used μ_ld acting on 4 days (as analysed in Fig. 4) since it better reflects the rapid isolation that happened during the first COVID-19 wave”, Line 369) and we corrected the labels in Fig. 9.

**Reviewer#2:** 

Evaluation
The paper has a number of shortcomings, and would require a much deeper discussion of several parts (see my main points). Also, the epidemiological language should be improved in many points. Some cited papers are weak. Therefore, the authors should (i) do an effort to (substantially) amend their paper according to the indications reported below, but especially (ii) put into evidence their findings that are of main epidemiological interest, particularly in relation to the insights that the adopted modeling framework would provide in relation to the understanding of COVID-19 control.

We thank Reviewer#2 for his/her careful comments and remarks.

Main points
The proposed SPQEIR model is quite restrictive in its formulation and therefore the underlying hypotheses should be carefully discussed. First, P individuals are – according to the stated hypotheses – fully protected, which corresponds to full segregation forever i.e., during the entire history of intervention measures. But measures aimed at confining susceptible people hardly can go beyond home confinement, and there is strong evidence that – during lockdowns - much COVID transmission occurred within households (especially during the first wave). Pairwise, also removed E individuals are fully inactivated in the SPEIR. Also, this should be discussed carefully.

We included these points for discussion and disclosed the model assumptions more explicitly (Sec 2.3 and Discussion, lines 473-485). In general, such points would indeed diminish the effectiveness of the control strategies, for which we assess the theoretical impact. We agree that including these aspects would likely make a significant difference on the exact values of the model variables during the epidemic waves. Nevertheless, we believe that they would not impact our take home message, i.e. that different measures impact differently the epidemic curves, but the same desired mitigation of the infectious curve can be obtained by social distancing plus synergies of other measures with potentially smaller socio-economic impact. We indeed proved this for 6 European countries. Fitting their data using either social distancing alone, or a synergy of strategies, results in milder social distancing (higher rho), thus assessing the generality of this finding.

Moreover, as E individuals can be removed mostly by way of tracing, it is not clear to me why you do not allow a pairwise option for I individuals (e.g. asymptomatic or pauci-symptomatic) by the same mechanism. Last, I understand screening and isolation of actively infected individuals is incorporated into removal (L99), which is an option. However, did you handle your removal rate to account for this (perhaps I didn’t note details)?

We agree with Reviewer#2’s remarks. The reason why we originally did not allow a pairwise option for I individuals, i.e. the possibility for I individuals to be removed from the infectious pool by quarantining, is that the first focus of the study was on investigating the role and the importance of preventive measures targeting S and E population groups, like tracing (e.g by app), protection of vulnerable individuals, and social distancing. Nevertheless, we fully agree that these neglect the fact that, once a person has become infectious (I), he/she might be tested and subsequently before having naturally recovered. Comparing the effect of this intervention with preventive ones is therefore very interesting. Hence, we modified the model, simulations and figures to incorporate this advice from reviewer 2. In particular, the ODEs are modified by an extra parameter \eta handling the removal rate, which in turn modifies R (Eq. 1). Then, we performed its systematic analysis in Sec. 3.1.4 and Fig. 7. The proposed synergies (Sec 3.2 and Fig. 8) now take \eta into account, as well as the fitting performed in Fig. 9. For fitting, we assumed by default that all countries worked to isolate contagious individuals; hence, the parameter \eta is associated to all. This is also disclosed in the description of Table 1. In other sections, the text was edited accordingly (Lines 151-154, 434-440).

L141 “We fit the model to the official number of currently infected (active) cases, for each considered country.” The authors are surely aware that published numbers of currently infectious cases poorly represent true infectious cases. So, this should at least be discussed more carefully. Moreover, this information risks of being severely biased when you aim at making comparisons between different countries – especially during the first wave - because it reflects the inter-country differences in testing and tracing, making comparisons unreliable.

We agree with the reviewer that published data of currently infected cases do not fully represent the true infectious cases, largely due to a fraction undetected cases, either because asymptomatic or because of lack of sufficient testing. Estimating this fraction of undetected can be based on prevalence studies based on antibodies tests, but ultimately this fraction remains largely uncertain. Thus, we decided not to employ such estimates in our model, as we considered the measured numbers of detected cases sufficient for the scope of our study, which is not to produce projections of the future evolution of the epidemic. Instead, it is intended to show how a synergy of measures could explain the observed curves of infection as nicely as social distancing alone, with lower levels of social distancing itself.

When choosing the countries for the subsequent analysis, we considered those with similar share of positive tests, aiming at excluding countries with too inefficient testing strategies and thus with overwhelmingly high numbers of undetected cases. We would like to stress that, while we do fit to 6 countries, our aim is not to compare the numbers of active infections between countries, but rather to verify our analysis of the effect of synergies on a number of real-world settings. Moreover, our focus is not on the values of active cases between countries, but rather on their dynamics, i.e. the shape of the infections curves. In addition, recent studies showed no statistically significant divergence of the data of the considered countries from theoretical trends [28,29]. Hence, it is reasonable to assume that these time-series data are capturing plausible time evolutions, which are thus informative of the epidemic dynamic.

In general, we acknowledge that Reviewer#2’s remark is particularly important for the overall interpretation of the results, so we broadened our discussion and reported the above discussion in the text (Lines 87-95 and 491-498).

L>140. Fitting procedures. The authors adopted a nonlinear least squares procedure citing a rather old textbook whereas the basic statistics of epidemic data has progressed dramatically in the last twenty years, first of all maximum likelihood techniques. For example, I do not understand how the quantity in (3) can be used to document the improvement in fit compared to the baseline model.

We acknowledge that most advanced inference techniques exist and are often employed in other frameworks. For instance, in a recent follow up study that our group developed in <https://www.medrxiv.org/content/10.1101/2020.12.31.20249088v1.full>, we employed Bayesian inference relying on Markov Chain Monte Carlo Methods to fit an extension of the present model. Here we considered the number of parameters small enough and the problem simple enough to justify the use of a simple non-linear least squares. Thus, the fitting procedure applied in this study relied on a non-linear Python pipeline that is in common use for problems with similar degrees of freedom as ours. To support that the method is enough for the goal, we can observe in Fig. 9 that the parameter values obtained allow to the model simulation to fit reasonably well the time-series data.

Finally, the reduced chi squared metric of eq.3 was not used for fitting, but to assess the goodness-of-fit, as it is a common diagnostic tool [Maydeu-Olivares, Alberto. "Maximum likelihood estimation of structural equation models for continuous data: Standard errors and goodness of fit." *Structural Equation Modeling: A Multidisciplinary Journal* 24.3 (2017): 383-394.; but also: Wen, Zhonglin, Kit-Tai Hau, and W. Marsh Herbert. "Structural equation model testing: Cutoff criteria for goodness of fit indices and chi-square test." *Acta psychologica sinica* 36.02 (2004): 186-194.]. The statistical justifications for this can be found e.g. in [43]. In fact, the chi square is the simplest possible form of the likelihood, obtained when writing the likelihood function assuming that errors on the measurement of the data are Gaussian distributed. In absence of better knowledge on the sources of errors on the available data, Gaussian distributed errors are the most common assumption in mean field models. An intuitive understanding can be gained as follows. The reduced chi square (3) is a quantity based on summing up the square distances of data-points from corresponding simulation values. It does provide a measure of the geometrical distance between the model simulation and the datapoints. Thus, smaller reduced chi square means smaller distance between model simulation and data, i.e. better fit. Thus, computing the quantity (3) for two models (e.g. the baseline model and the model with synergies) and comparing the two obtained numbers allow to asses which of the two models provide a better fit to the data.

L83 “the time T passed until no new infections occur”, this is quite wrong at least as far as your model is a deterministic one, as I understand it is. In the practice of simulation this does not need to be a problem (and indeed you acknowledge this at a later stage), but the sentence should be modified.

We agree, thus the sentence has now been modified (Line 108).

L119, the effective reproduction number is not explicit in the SIR model, because the susceptible fraction is not explicit. Therefore, the formula drawn from ref [26] is an approximation. The problem is that it is far from being general and rests on a number of hypotheses, which I find somewhat naive. This also holds for eq (1). I noted that even in the cited paper [26] the formula is given without a justification. The formula trivially holds if you assume that the removal by segregation of the susceptible population occurs rapidly, that is before the susceptible population is sufficiently depleted. In this case S(t) = Nexp(-\μt), so that if you additionally assume that \μt is small (which contradicts that segregation occurs fast) and resort to the linear approximation of the exponential function you arrive to the point. Anyhow, is this relevant for this paper? On top, I recommend to avoid to cite whatever paper appeared in this epoch because the quality is not necessarily good (sometimes poor) and may induce errors in readers.

We agree with Reviewer#2’s analysis and his/her remark that, in fact, an explicit dependence to mu does not hold in case of hard lockdown (e.g Fig. 4) and does not add value to our analysis. Hence, we edited the text accordingly (line), by avoiding citing poorly supported papers and by editing eq. 1 in general terms that are not subject to the mentioned assumptions. The subsequent analysis is performed following the updated eq.1.
As for the remark about recent literature, we agree about being careful when citing non-peer reviewed works from the latest months. We amended our bibliography in this respect.

Legends of simulation exercises are scanty and should be improved.

We improve the legends in simulation and fitting figures.

Other points
> L34, “homogeneous propagation media” is naïve for most readers of the Journal; as epidemiologists we speak of “a homogeneously mixing population” which is a nowadays somewhat universally agreed terminology.

The text was edited (Line 47).

IBM models are not continuous but discrete models (due to their very structure of simulative models)

The text was edited (Line 52).

L42, "likely" : socially active and at risk of infection

The text was edited (Line 55).

L83 Clearly, the SEIR model by its I curve provides only a very indirect measure of the pressure on PH system (instead represented by ICU and hospitals occupancy). Even more so for the economic system. This should be discussed.

We edited the text to avoid overstatements about possible direct influences of the I curve on other systems (Lines 106-109), and we discussed and supported its relevance in epidemic management by referring to multidisciplinary studies [17, 18, 31-34]. We did not include such aspects in the current model because they went beyond the question we tackle here.

L112 “resulting in the effective reproduction number”, In epidemiology the effective reproduction number deals with a situation where the susceptible proportion is depleted below one, as you correctly say in the subsequent line. Suggest to rephrase.

The text was edited (Lines 141-144).

L126 “We use mean values etc”, please clarify

The text was edited (Line 155).

L129 “with conservation of the total number of individuals, meaning N’ = 0”, the argument goes the other way round, your system fulfills N’=0 implying that N is conserved.

The text was edited (Line 160).

L86 “Mainstream suppression measures against the epidemic aim at flattening the curve of new infections”, replace “suppression “ by “mitigation” (flattening the epidemic curve is somewhat different from suppressing)

The text was edited (Line 112 and throughout the text).

L161 “comparative information analysis” this is not an agree terminology

The text was edited (Line 193-196).

Minor points
I suggest to delete “new” from the abstract and simply state: “an extended SEIR model including quarantine of susceptible an latently infected individuals”

We agree about this and edited the text accordingly.

L8 “statistical methods allow for accurate characterization of the population's health state", stated like this is a bit trivial.

The text was edited (Line 8).

L111 “repression” not appropriate

The text was edited (Line 140 and throughout the text).

L117 “physical reduction of a country's population” ?

We meant that the active population within a country could diminish after reduced commuters’ activity. For instance, this happened and had a great impact in the country of our institution (Luxembourg). The text was therefore edited (Line 146).

L182 I suggest to replace “mathematical” in the title with “Simulation”: it is a simulative analysis

The text was edited (Line 214).

L189 “eradication time” inappropriate wording

The text was edited (Line 221).

L194 Citation 32 is not appropriate. That paper considered a model with behavioral responses which are not included here.

We agree with Reviewer#1 and we removed the reference not to confuse the reader and edited the text accordingly (Line 224).
