# Supplementary material for: Dynamical SPQEIR model assesses the effectiveness of non-pharmaceutical interventions against COVID-19 epidemic outbreaks
Source: PLoS One. 2021 May 21;16(5):e0252019. doi: 10.1371/journal.pone.0252019 (PMC8139462; doi:10.1371/journal.pone.0252019)
Supplement: Attachment — Submitted filename: response_to_reviewers.docx [file pone.0252019.s004.docx]

Dear editor, dear reviewers,

We are glad that Reviewer #2 is entirely satisfied by our revision, and we thank Reviewer #1 for the list of suggested improvements, which encouraged us to significantly extend our work, and making it more complete. Please find below detailed responses to the points that were raised. Our responses are in blue. In the main text, the parts that we edited based on comments from Reviewer #1 are in red. Following Reviewer #1 suggestions, we performed the analysis of further interesting aspects that complement the main findings reported in the manuscript, and we created a Supplementary Information S1 Text to discuss those additional analysis.

Reviewer #1: The authors now clarify the main claim of their work: "We ultimately show that analogous containment levels of the infectious curve can be achieved by alternative synergies of non-pharmaceutical interventions." So the message of the paper now is clear and one can better evaluate the results. However several important points in my opinion still require improvement.

The authors claim that similar results can be obtained with different interventions. Figure 8 indeed provides similar curves for the first 4 scenarios where the value of the effective reproductive number is similar, while the evolution of scenarios 5 and 6 is different but these scenarios present a different effective R. In this perspective one could infer that scenarios with similar reproductive number display similar epidemic evolution, which is not surprising. Can you discuss this point?

The point raised by Reviewer #1 is correct. It is indeed known that epidemic dynamics are driven by the control parameter R_eff. However, there are multiple ways to achieve the same R_eff. Indeed, R_eff results from different combinations of finer-gained parameters. Figure 8 shows how to achieve fine-tuning of different interventions by acting on specific parameters, which could eventually be lumped into the global R_eff as explained in Eq. 1. While Fig. 8 displays a limited number of example scenarios, others can be simulated with our companion Shinyapp (https://jose-ameijeiras.shinyapps.io/SPQEIR_model/). Following Rev. #1’s suggestion, we discussed this point further in the text (Sec. 3.2).

Moreover, you fit the data set using as fitting parameters only social distancing or considering all the parameters of the non-pharmaceutical interventions. Similar quality of the fittings are obtained, evidencing that different approachess can be adopted. However, the fit with the whole parameter set is very similar to the case where only social distancing is present (the parameters rho are close, while mu_ld chi and eta are very small). Therefore one could suppose that social distancing is the main intervention observed in the data.

The fitting called “only social distancing” is primarily performed to assess a reasonable value for R_eff that is less sensitive to the identifiability issue (fitting degenerate parameters) discussed in the text. This is done to make a comparison with the R_eff value obtained with fine-grained parameters fitting. Indeed, we observe that social distancing plays an important role even for the other combinations of interventions, which is not surprising as it is the main population-wide intervention during European-like lockdowns (as also reported in Table 1), while others are more targeted to individuals. Nonetheless, other parameters, despite being low, induce non-negligible impacts (for instance, a moderately high number of individuals flow into the outer compartments, as reported in Table1 of SI Text). We discuss this impact in the main text (Sec. 3.3 and 4) as well as in the new Supplementary Information SI Text (SI Sec. 2), which was added for this purpose.

What happens e.g. if you consider in the fit only a lock down, which has been a common intervention in the first wave of COVID 19?

Our strategy was based on fitting only those parameters that are associated with interventions observed and reported in the considered countries (as discussed in Table 1 of Main Text). Hence, we did not perform different fitting with alternative parameters combinations, that were instead not observed in reality (cf. ACAPS database reference in Main Text). As this goes beyond the scopes of the present manuscript, further studies might investigate these aspects. To improve the discussion, we expanded this point in the text (Sec. 3).

You state that important correlations among the fitting parameters is observed. It should be interesting if you are able to show what is the region in the parameter space (rho,mu_ld, eta) where you obtain a nice fitting of the epidemic curves with a similar (small) value of chi^2. In this way one could compare the relevance of the different possible interventions. According to the previous hypothesis this region should correspond to a region at constant value of the reproductive number.

The identifiability of epidemiological parameters is indeed an interesting point. To better explore the parameter space, we expanded our previous analysis, that was based on a gradient descent non-linear fitting from the lmfit python library. The new extension of our analysis was performed with a Bayesian Inference framework based on Markov Chain Monte Carlo (MCMC) methods. This allowed a better exploration of the parameters space, highlighting regions corresponding to similar R_eff values and similar fitness to data. This additional analysis is now included in the new Supplementary Information SI Text (SI Sec. 2). Figs. 5-7 outline the areas of parameter space (for different couples of parameters and different countries) corresponding to a high posterior probability, and thus a good fitness of the model to the data.

A small constant value of mu as modelling of protection in my opinion is not realistic. The case where a fraction of the population isolate in a small time well represent a lock down. While a constant rate of isolation during the whole epidemics it seems to me that do not represent a realistic non pharmaceutical intervention. While people should self isolate with a small constant rate? On the other hand, a constant mu could well represent vaccination which is not a pharmaceutical intervention. Moreover, the sentence: "In particular, μ = 0.01 d −1 ..... through isolation, this is unrealistic." seems to be in contrast with the large value of mu of figure 2.

We agree with Reviewer #1’s comment and we edited the considered sentences accordingly.
In general, small \mu values are investigated in their corresponding section to complete the conceptual analysis, but only high values during a short time were used to perform the country-wise fitting, thus mimicking the beginning of a lockdown in Europe and reproducing the behavior discussed in Fig. 4.

In the lock down description where a large value of mu is activated for a small time to isolate quickly a finite fraction of the population I do not understand why only susceptible people are put into quarantine. I expect that a general measure as a lock down involves all the population including also exposed and infected individuals. Indeed the aim of a lock down is not only protection of the susceptible nodes but also isolation of potentially infected people.

This is indeed an important point. From a modelling perspective, all outfluxes from the main epidemic compartments (framed in Fig.1) to Q and P compartments correspond to isolating measures. During the whole intervention period, all outflux parameters are active, thus modelling the observed interventions that were applied during European-like lockdowns, which also targeted potentially infected people as explained in Table 1.
On the other hand, we named the intervention parameters after the compartment they act upon. So the action of “protection” is on susceptibles only, whereas the removal of individuals from the infectious compartments (framed in Fig.1) comes from the combination of all outflux parameters.

10 days from the onset of the first contagion is an arbitrary choice. The importance of an early adoption of measure of containment is a well known fact. Clearly 10 days belongs to such early adoption framework what happens if measures are taken after a longer time. The different strategies are still equivalent?

We agree with the reviewer that 10 days is to some extent arbitrary. This aspect is indeed of interest for readers, as the delay \tau represents an extra parameter that might influence the dynamics. To answer in detail and to improve the content of our manuscript, we performed additional simulations and included an extended discussion section in the Supplementary Information SI Text (SI Sec. 1). We observe that the qualitative evolution (trends, dependence on parameters, etc.) of the epidemic curve under interventions does not change, and its quantitative details (height of the peak, absolute number of days until mitigation) change according to Figs. SI 1-4. Of particular interest is the resulting tradeoff of prompt-but-weak and delayed-but-strong interventions, which cause different quantitative outcomes. Interestingly, such outcomes are currently observed in several European countries.

I do not understand the sentence: "However, we notice that values of ρ ' 0.3 or lower are more effective in mitigating the epidemic faster.?" clearly the smaller is rho the more is effective the measure to contain the epidemics, but what is the relevance of this comment?

In Fig.2c of main text, we highlight a non-monotonous and, more intriguing, non-linear relationship between the peak height (and the mitigation timing) and the intervention parameters. The aforementioned sentence was intended to stress this point, and the fact that there are certain parameter values that minimize the peak and the timing. However, we acknowledge that it was initially not entirely clear, and we now edited the text accordingly.

We thank Reviewer #1 for his/her feedback, which led us to perform further analysis and increase the depth of the manuscript.
