# Supplementary material for: Dynamical SPQEIR model assesses the effectiveness of non-pharmaceutical interventions against COVID-19 epidemic outbreaks
Source: PLoS One. 2021 May 21;16(5):e0252019. doi: 10.1371/journal.pone.0252019 (PMC8139462; doi:10.1371/journal.pone.0252019)
Supplement: Attachment — Submitted filename: response_to_reviewer.docx [file pone.0252019.s005.docx]

Dear editor, dear reviewers,

Once again, we thank the reviewers for their further suggestions and improvements. Please find below detailed answers to the questions raised, and explanations on how we tackled the points specified by reviewer #1. Our responses are in blue. In the main text, the parts we edited following your suggestions are in red.

Reviewer #1: The authors have answered to my previous questions and in general the paper is improved accordingly.
However some points still require an improvement:

My comment on the fact that during a lock down not only susceptible individuals are removed but also infected and exposed, has not been addressed clearly:
"This is indeed an important point. From a modelling perspective, ........the
combination of all out-flux parameters" If this means that during the short period of isolation of individuals (4 days) you set mu=mu_ld but also eta=mu_ld and xi=mu_ld so that also exposed and infected individuals are removed with the same rate?
In my opinion this should be a correct approach: during a lock down the same fraction of S, E and I individuals are typically protected. If this is the case it should be explained, otherwise if you set only mu=mu_ld, the model isolate only susceptible individuals during a lock down which is not very likely and some explanations should be added.

We fully agree with Reviewer #1 that the suggested fitting scheme is more realistic. Hence, following the reviewer’s suggestion, we have now tested both methods (mu=mu_ld, as we employed so far, and all parameters=mu_ld during the initial 4 days of rapid isolation). While the latter is more realistic, as mentioned, the “mu=mu_ld” method is better connected to our conceptual analysis. Thus, testing both methods give an indication if a more complex model improves the goodness of fit.
Hence, we performed the fitting to country data with one method at a time, thus obtaining two sets of fitted parameters rho, mu_ld, eta etc., like in Fig.9. Our results confirm that, during the first epidemic wave, the two approaches yield similar quantitative results, as the fitted parameters differ only in the second significative digit while the reduced chi-squared metrics gets slightly worse. Thus, we can conclude the two approaches reproduce the epidemic dynamics equally well, leaving our conclusions unaltered. For illustration, we report fitting values for some example countries. Others display similar subtle changes.

Denmark

| **Method** | rho | mu_ld | chi’ | eta | R | chi2red |
| --- | --- | --- | --- | --- | --- | --- |
| mu_ld acting on S | 0.35 | 0.003 | 0 | 0.0014 | 0.73 | 3.8864e-09 |
| mu_ld acting on all compartments | 0.36 | 0.019 | 0 | 0.0013 | 0.73 | 6.2272e-09 |

Israel

| **Method** | rho | mu_ld | chi’ | eta | R | chi2red |
| --- | --- | --- | --- | --- | --- | --- |
| mu_ld acting on S | 0.31 | 0.001 | 0.001 | 0.004 | 0.7 | 1.6054e-08 |
| mu_ld acting on all compartments | 0.30 | 0.001 | 0.001 | 0.001 | 0.7 | 4.4888e-08 |

Switzerland

| **Method** | rho | mu_ld | chi’ | eta | R | chi2red |
| --- | --- | --- | --- | --- | --- | --- |
| mu_ld acting on S | 0.28 | 0.001 | 0 | 0.001 | 0.67 | 6.0977e-09 |
| mu_ld acting on all compartments | 0.276 | 0.001 | 0 | 0.001 | 0.66 | 6.9493e-09 |

This is due to the fact that, at the beginning of the pandemic, the S compartment contains by far the greatest number of individuals (since S~N); hence, parameters related to the S compartment have a larger impact on the infection dynamics. In addition, the overall impact of mu_ld is smaller than that of other parameters, such as rho, because of the nature of European-like lockdowns. In fact, the variable P in our model, controlled by the parameter mu_ld, represents the compartment of individuals with zero probability of getting infected, which correspond to a minority of people, e.g. to elderly people fully isolated and not the majority of individuals who had still some external contacts due to e.g. going to work, having a family member going to work, doing the groceries etc. The effect of the lockdown on these individuals is represented in our model by the parameter rho, which describes a decreased (but not an absence) level of social interactions.
Following Reviewer#1, we edited the text (lines 386-389) to make this point clearer.

The authors introduce, in the supplementary, the analysis of the posterior probability distribution for different values of the parameters, evidencing that a nice fit of the experimental data is obtained in a wide region of the parameter set. This nice point answer to one of my previous question. However, I do not understand in figures 5,6 and 7 when you plot the probability as a function of two of the parameters how do you fix the value of the other parameters which are not consider in the plot.

We now realised this part was not clearly explained in the text. An important point here is that the parameters are not statistically independent. The posterior distribution for a particular parameter depends on the values of other parameters. Hence, to visualise a joint posterior distribution of 2 parameters from a high dimensional one, we need to first marginalise it against the other parameters. In probability, marginalising corresponds to projecting high dimensional distributions to the dimensions of interest by integrating over the remaining (“marginalised-out”) parameters. As an example, taken from <https://en.wikipedia.org/wiki/Marginal_distribution>, to obtain the marginal probability of *x* from the joint probability distribution of *x* and *y* we would take

$$p_{X}\left( x \right)= \int_{y} p_{X|Y}\left( x|y \right) p_{Y}(y)dy$$

This can also be exemplified by the following figure (CC0, taken from <https://en.wikipedia.org/wiki/Marginal_distribution>), where a 2D distribution (highlighted by the green ellipse) is marginalised over *X* (leading to the 1D red *p*(*y*) distribution) and *Y* (leading to the 1D blue *p*(*x*) one), respectively.

Similarly, we marginalised the full distribution obtained by MCMC chains to produce Figures 5, 6 and 7 of Supplementary Information. We added the word *marginal* to clarify this point, and discussed it further in Sup Mat (end of Sectioon 2.2. of Supplementary Information).
Finally, it has been reported (cf. Table 1) that some countries did not employ certain strategies. Thus, for those countries, the corresponding parameters were fixed to default values as described in Sec. “The extended SPQEIR model to reflect mitigation strategies”.

There is a typo at page 12: "Sec. ??"

The typo has now been corrected, we thank Reviewer#1 for noticing.
